# Elucidating the Potential Inhibitor against Type 2 Diabetes Mellitus Associated Gene of GLUT4

**DOI:** 10.3390/jpm13040660

**Published:** 2023-04-12

**Authors:** Afaf Aldahish, Prasanalakshmi Balaji, Rajalakshimi Vasudevan, Geetha Kandasamy, Jainey P. James, Kousalya Prabahar

**Affiliations:** 1 Department of Pharmacology, College of Pharmacy, King Khalid University, Abha 61421, Saudi Arabia; adahesh@kku.edu.sa (A.A.); raja@kku.edu.sa (R.V.); 2 Department of Computer Science, King Khalid University, Abha 61421, Saudi Arabia; 3 Department of Clinical Pharmacy, College of Pharmacy, King Khalid University, Abha 62529, Saudi Arabia; glakshmi@kku.edu.sa; 4 Department of Pharmaceutical Chemistry, NGSM Institute of Pharmaceutical Sciences (NGSMIPS), Nitte (Deemed to be University), Deralakatte, Mangaluru 575018, Karnataka, India; jaineyjames@gmail.com; 5 Department of Pharmacy Practice, Faculty of Pharmacy, University of Tabuk, Tabuk 71491, Saudi Arabia; kgopal@ut.edu.sa

**Keywords:** T2DM, GLUT4, ADMET, CoFACTOR, ChEMBL, MOE

## Abstract

Diabetes is a chronic hyperglycemic disorder that leads to a group of metabolic diseases. This condition of chronic hyperglycemia is caused by abnormal insulin levels. The impact of hyperglycemia on the human vascular tree is the leading cause of disease and death in type 1 and type 2 diabetes. People with type 2 diabetes mellitus (T2DM) have abnormal secretion as well as the action of insulin. Type 2 (non-insulin-dependent) diabetes is caused by a combination of genetic factors associated with decreased insulin production, insulin resistance, and environmental conditions. These conditions include overeating, lack of exercise, obesity, and aging. Glucose transport limits the rate of dietary glucose used by fat and muscle. The glucose transporter GLUT4 is kept intracellular and sorted dynamically, and GLUT4 translocation or insulin-regulated vesicular traffic distributes it to the plasma membrane. Different chemical compounds have antidiabetic properties. The complexity, metabolism, digestion, and interaction of these chemical compounds make it difficult to understand and apply them to reduce chronic inflammation and thus prevent chronic disease. In this study, we have applied a virtual screening approach to screen the most suitable and drug-able chemical compounds to be used as potential drug targets against T2DM. We have found that out of 5000 chemical compounds that we have analyzed, only two are known to be more effective as per our experiments based upon molecular docking studies and virtual screening through Lipinski’s rule and ADMET properties.

## 1. Introduction

Diabetes is a chronic hyperglycemic disorder that leads to a group of metabolic diseases [1]. This condition of chronic hyperglycemia is caused by abnormal insulin levels. The impact of hyperglycemia on the human vascular tree is the leading cause of disease and death in type 1 and type 2 diabetes [2]. Similarly, people with type 2 diabetes mellitus (T2DM) have abnormal secretion as well as the action of insulin [3]. Type 2 (non-insulin-dependent) diabetes is caused by a combination of genetic factors associated with decreased insulin production, insulin resistance, and environmental conditions. These conditions include overeating, lack of exercise, obesity, and aging. Insulin insensitivity, a decline in beta-cell activity, peripheral insulin resistance, and impaired control of hepatic glucose production are all symptoms of T2DM, which eventually leads to the failure of pancreatic beta-cells [4]. Inflammatory reactions can cause a causal association in the development of T2DM, increasing insulin resistance or they can enhance hyperglycemic conditions leading to T2DM [5]. Chronic low-grade inflammation and activation of the immune system have a role in the insulin resistance and pathogenesis of this disease [6]. It has been reported that type 2 diabetic and insulin-resistant animals or patients have abnormally high levels of oxidative stress mediators or pro-inflammatory cytokines [7].

T2DM is a progressive and complex disorder marked by a variety of metabolic abnormalities that affect a number of organs. The risk of developing T2DM is directly related to low levels of inflammation. Through insulin resistance, inflammatory reactions contribute to the development of T2DM. Insulin resistance may be increased and lead to long-term problems in the presence of hyperglycemia. By studying the link between the development of T2DM and inflammatory biomarkers, it has been shown that adipose tissues release tumor necrosis factor-alpha and compounds after completing fatty acid production. Inflammatory factors are directly linked to body fat levels [2].

Glucose transport limits the rate of dietary glucose used by fat and muscle. The glucose transporter GLUT4 is kept intracellular and sorted dynamically, and GLUT4 translocation or insulin-regulated vesicular traffic distributes it to the plasma membrane. One of the most important functions of insulin is to stimulate glucose absorption in fat and muscle tissues, which is necessary for energy storage. It was identified that a shift in the distribution of glucose transporters from intracellular storage vesicles to the plasma membrane of rat fats and muscles causes an increase in the uptake of glucose. The rate of glycemic glucose utilization by muscle tissues is limited by glucose transport, and GLUT4 translocation is decreased in type 2 diabetes [7]. As glucose transfer across the cell membrane is a rate-limiting step in glycolysis, glucose transporters are crucial in tumor growth and development.

GLUT4 solute carrier family 2 member 4 (SLC2A4) gene [8], which encodes glucose transporter 4, is a candidate gene for type 2 diabetes mellitus (T2DM) [9] and also a potential target in cancer treatment [10]. GLUT4 (SLC2A4) gene is the insulin-responding glucose transporter, found predominantly in muscle cells and adipocytes (fat cells) [11], and plays an important role in glucose regulation. Biochemical characteristics, tissue-specific expression, and physiological roles all work together to regulate as well as maintain the level of glucose and its distribution in the GLUT family. Its expression has been shown to affect glucose metabolism and tumor generating. GLUT4 is the most important GLUT in glucose metabolism, accounting for 90% of all GLUT [10].

Repositioning or repurposing drugs accounts for a substantial part of entering approval pipeline drugs, which indicates that drug repositioning has huge market potential and value [12]. Drug repurposing is successful in finding treatments for various diseases, especially for type 2 diabetes mellitus (T2DM) [13]. The major classes of antidiabetic medications for T2DM are difficult to reach a reasonable balance in the role of efficacy, side effects, and patient tolerance. Hence, drug repositioning provides a cost-effective and promising approach to finding new effective drugs for T2DM. Computational technologies have accelerated the process of drug repositioning in the last few decades years. Virtual screening has reached a status of a dynamic and lucrative technology in probing for novel drug like compounds or so-called hits in the pharmaceutical industry [14].

Secondary metabolites comprise a wide range of chemical substances such as polyphenols, organo-sulfur compounds, steroidal saponins, alkaloids, and flavonoids. Despite the fact that the majority of these compounds are naturally synthesized, their synthesis can be augmented under stress situations depending on the development environment and stresses [15]. These molecules play a vital role in the prevention of diseases such as diabetes, liver disease, cancer, and cardiovascular disease. These compounds, when consumed, can have a stronger impact on epigenetic alteration, which is crucial in cancer prevention and treatment [16].

## 2. Materials and Methods

### 2.1. Protein Selection and GLUT4 Sequence Retrieval

The *GLUT4* gene was selected on the basis of its high expression in type 2 diabetes mellitus; it is observed that the overexpression of this gene activates the metabolic pathway for insulin shortage and glucose deposition in the blood stream which ultimately leads to T2DM. The nucleotide sequence for this gene is retrieved from GenBank NCBI Database [17] (https://www.ncbi.nlm.nih.gov/genbank/ accessed on 5 February 2022), and the protein sequence for this protein was retrieved from the UniProt knowledge database with accession number P14672 [18] (https://www.uniprot.org/ accessed on 5 February 2022).

### 2.2. Phylogenetic Analysis and Motif Detection

After retrieving the gene sequences, a customized BLAST [19] search was executed to check and identify both inter-species and intra-specie *GLUT4* gene variants. After that, multiple sequence alignment was performed using CLUSTAL-OMEGA [20] to observe highly conserved regions among those variants, which lead to conserved domain identification. MEGA-7 was used for phylogenetic analysis to trace back the evolutionary pathway of gene *GLUT4*, maximum likelihood method with bootstrap value 500 was used and executed for this purpose. After constructing a phylogenetic tree MEME server [21] (https://meme-suite.org/meme/ accessed on 5 February 2022) was used to find the motifs in this gene.

### 2.3. Protein 3D Structure Prediction and Evaluation

Protein 3D structure for glucose transporter glut4 protein was predicted using different approaches, such as homology modeling, threading, and ab initio to perform comparative modeling. Homology modeling was performed using MODELLER, which is offline software. MODELLER techniques perform comparative modeling by deriving the alignment of the target sequence with the structure of the template. The restraints and CHARMM energy terms are combined into an objective function. Restraints are obtained from protein structure alignments. Then, the resulting model was found by adjusting the objective function by employing methods of conjugate gradients and molecular dynamics with simulated annealing, as per the standard MODELLER protocol [22].

To obtain the appropriate template, the BLASTp analysis against PDB was performed using a query sequence. SWISS-MODEL was used to predict the accurate 3D structure of the protein which uses the homology modeling approach. QUARK was used for threading 3D structure prediction. Raptor X was also used for protein structure prediction. Predicted protein structures were evaluated using RAMPAGE, Verify3D, ERRAT, What-Check, Molprobity, and Prove. Different parameters of the predicted proteins were checked with different tools.

### 2.4. Binding Site Prediction

An active binding region is always very important for successful molecular docking analysis. The binding site of the protein was predicted using COFACTOR software. Multiple physiochemical properties. i.e., molecular weight, half-life, toxicity and residual proportion of the glut7-glucose transporter were evaluated using the ProtParam Expasy server accessed at https://web.expasy.org/protparam/ accessed on 5 February 2022.

### 2.5. Virtual Screening and Molecular Docking Analysis

A library of 5000 chemical compounds was created by obtaining phytocompounds from different open-source databases such as SBL and PubChem. The database needs to undergo several filtering processes to decrease the huge number of compounds. The phytocompounds were downloaded using filters such as “druglike”, and “in-stock”, and selection was based on Lipinski’s rule of five. These compounds were selected and then drawn using Chem3D ultra. Molecular docking analysis of the GLUT4 glucose transporter was performed against the optimized library of chemical compounds. Molecular operating environment (MOE) software version 2019 [23] was used to perform molecular docking analysis. Docking analysis was completed using default parameters, as the force field was set to MMFF94x and used London dG method for the posing and scoring process [24].

Ligand placements were assessed with the root-mean squared-deviation (RMSD) between the heavy atoms of the predicted pose and those of the crystal structure. The percent success (% success) for placement was defined as the number of systems where the RMSDs to the crystal structure of a docked pose is less than a given threshold. On the basis of the root mean square deviation (RMSD) value and S-score, best results were selected. After docking analysis, the interactions between protein atoms and ligands were checked using CHIMERA.

### 2.6. Physicochemical and ADMET Properties

The selected hits were then further screened using admetSAR v1.0 [25] and PreADMET v2.0 [26] servers to evaluate the absorption, distribution, metabolism, excretion, and toxicity (ADMET) properties. The physicochemical properties, Lipinski’s RO5 violations, and other parameters such as percent human intestinal absorption (HIA), degree of plasma protein binding (PPB%), blood-brain barrier penetration (BBB), Madin–Darby canine kidney (MDCK), and Caco-2 cell permeability were considered. In addition, toxicity properties were also checked.

### 2.7. Prediction of PAINS by Promiscuity Assessments

Pan-assay interference compounds (PAINS) are promiscuous molecules with multiple behaviors that interfere with assay readouts. They are compounds showing non-target specific activity in high-throughput screening that can mislead medicinal chemists during hit identification, wasting time and resources [27]. The promiscuity of six compounds was determined by the online program Hit Dexter 2.0. [28,29] and SwissADME [30]. The SMILES (simplified molecular input line entry specification) format was entered into the input box, and the results were generated automatically.

## 3. Results

### 3.1. Database Search, Comparative Phylogeny, and Physiochemical Properties Prediction

The GLUT4 gene sequence was retrieved from NCBI, then BLASTx, NCBI was used to extract protein sequence. The protein sequence was also retrieved from UniProt, and then both sequences were compared in order to check any difference, but both sequences were similarly curated. Physiochemical properties of the protein sequence were evaluated using ProtParam tool.

**Figure 1:**
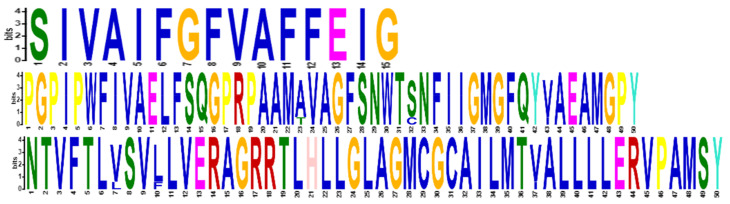
Multiple sequence alignment analysis of GLUT4 protein or transporter from different species showing most conserved domains involved in sugar transport.

In addition, the BLAST tool was used to retrieve the same gene sequence from different organisms including chimpanzee, gorilla, rhesus macaque, crab-eating macaque, and lion as an out-group. All sequences were aligned globally to perform MSA using MEGA7. It is observed all sequences share a common domain (Figure 1), although the GLUT4 gene from the lion has a slightly different sequence.

**Figure 2:**
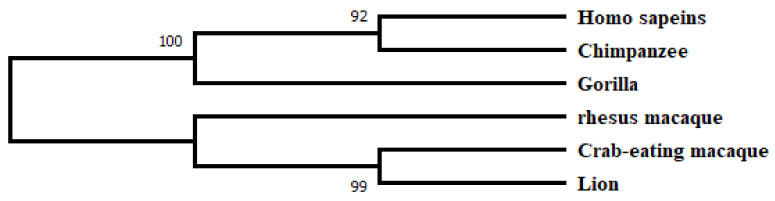
Phylogenetic analysis of *GLUT4* genes from different species showing evolution pattern evaluated using MEGA7, maximum likelihood method at 1000 bootstraps.

Then, phylogenetic analysis is performed, and a tree is constructed using MEGA7 maximum likelihood method. It is observed that GLUT4 from Homo sapiens shares a common clade with chimpanzee, which means both are more closely related as compared to any other species under consideration. The lion is at a maximum distance from our query gene, which confirms the accuracy of our experimental run (Figure 2).

### 3.2. Structural Modeling, In Silico Characterization, and Model Validation

**Figure 3:**
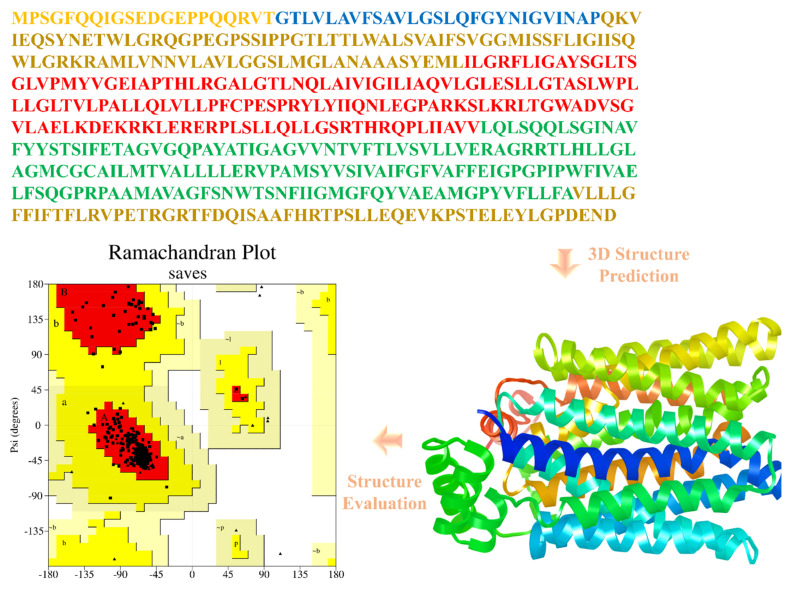
Finally selected GLUT4 protein structure based upon best quality factor and its sequence and Ramachandran plot showing its integrity and accuracy.

The protein was modelled through the SWISS-MODEL model server using the method homology modeling. The template was selected based on the query coverage and the structural identity of the query sequence 97% and 62%, respectively. Furthermore, the modelled protein was validated through the Ramachandran plot, and it shows 97% of the residues are in the most favored region, clearly showing that the model was built properly and was taken to the molecular docking studies. The visualization module of the UCSF chimera was used to visualize the predicted models. The evaluation of all predicted models was performed for their stereo-chemical quality assessment. Additionally, to check the stability, reliability, compatibility, quality, and accuracy of the computationally predicted structure of the protein, a comparative study with experimentally solved structures of crystal was performed in each case of qualitative assessment. The RAMPAGE server used to obtain the Ramachandran plot of predicted model GLUT4, which predicted % residues (97.1% in most favored and 2.6% in additionally allowed region and 0.3% in generously allowed region clearly shows that the model was a high-quality 3D structure taken to further studies. (Figure 3).

### 3.3. Principal Cavity Prediction

**Figure 4:**
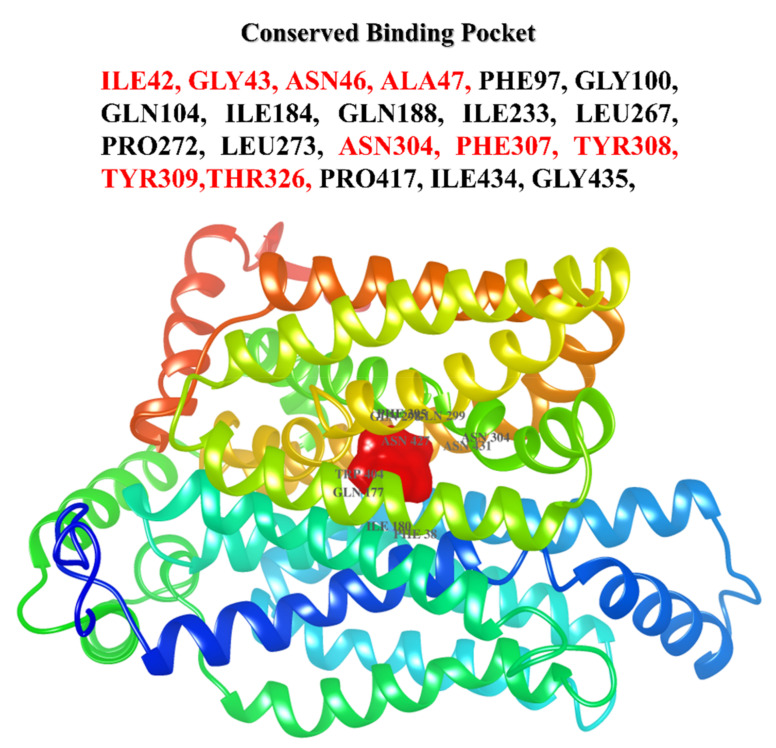
Active site prediction: Binding residues which are involved in the attachment of active compounds against GLUT4 protein for its better functioning. Dense red color at the center of the protein structure shows the attached possible active compounds based upon previous experiments by other scientists.

CoFACTOR server was used to predict minor and major binding sites of the putative ligand for the predicted protein. Specificity was provided from c-terminal residues for phytochemical binding containing conserved active site residues such as ILE42, GLY43, ASN46, ALA47, PHE97, GLY100, GLN104, ILE184, GLN188, ILE233, LEU267, PRO272, LEU273, ASN304, PHE307, TYR308, TYR309, THR326, PRO417, ILE434, and GLY435 compared to the residues of the active site for the template. In the predicted model GLUT4, the binding site of other substrates revealed the extensive conservation of tyrosine, serine, and isoleucine residues ILE184, GLN188, ILE233, LEU267, and PRO272. The residues of glycine were observed to be conserved at different positions in the GLUT4, which represent their vital role in phytochemical binding including GLY43, ILE184, ASN304, PHE307 represented by ILE42, ASN46, ILE184, ASN304, PHE307, and TYR308 which may play a crucial role in the specificity of GLUT4 (Figure 4).

### 3.4. Molecular Docking Results

ChEMBEL and ZINC databases were used to retrieve the library of 5000 active compounds. The docking was performed between the modeled protein GLUT4 and 5000 active compounds to generate their binding mode. To refine the best pose, the dynamic simulations were performed with allowed conformational change in modeled structure of GLUT4. CHIMERA, MOE, PyRx were used for the evaluation of protein and phytochemical interaction. The software showed variations in outcomes for correct pose prediction among the many possible docking poses, as indicated by scoring functions, which may lead to the conclusion that docking scores are not exact enough to represent protein-ligand binding affinity.

Moreover, Chimera was used to analyze the molecular docking to measure the dissociation constant (kd) μM and docking score (kcal/mol). The virtual screening and molecular docking using ADMETsar, MOE, and PyRx ranked the ligands for each complex, which based on a precise prediction of pose (the potential of ligand to bind for a certain conformation of the receptor) in order to differentiate those ligands that do not bind in a ranked list. The top six ligands were selected for molecular docking studies to study their binding interactions, which are listed in Table 1.

### 3.5. Physicochemical and ADMET Properties

The observations in Table 2 exhibit the physicochemical properties, which includes molecular weight, logP, hydrogen donor, and acceptor. The Lipinski’s rule of five predicts that there were no violations.

In molecular docking studies, two compounds show the hydrogen bond interaction with the protein target, which indicates that both molecules are highly selective, and the salt bridge interaction depicts a stronger interaction with the target as shown in Table 3. The residues ASN304 and ASN431, ASN 333 form the conventional hydrogen bonds, and GLU396 form the carbon-hydrogen bonds, and the salt bridge with the target, and these two compounds could be a good inhibitor against the selected protein targets (Table 2).

**Figure 5:**
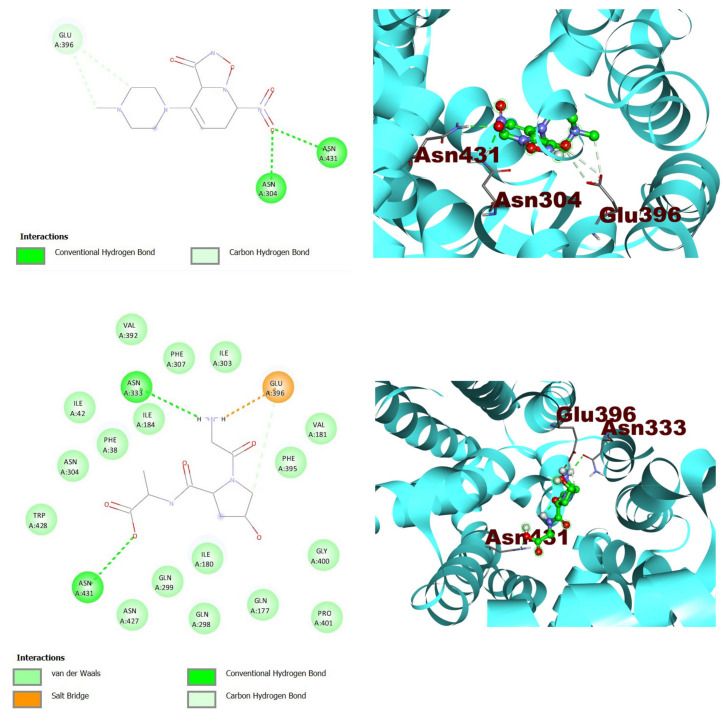
A molecular docking interaction analysis between the two most suitable active chemicals: 2D-interaction diagram (**left**) 3D-interaction diagram (**right**) for ZINC000216155214 and ZINC000001576020, against GLUT4 protein.

After the interactions of protein chemical compounds, the molecular complexes have been visualized using Discovery Studio Visualizer (Figure 5).

In Table S1, the absorption values (HIA, BBB, PPB, PCaCO2, and PMDCK) predicted for the compounds can be observed. Ligands ZINC000001643171, ZINC000017064359, and ZINC000216155214 have good HIA (i.e., 86.25%, 83.47%, and 79.39%, respectively). All the compounds taken for the study were found to have moderate HIA. All the compounds under study had CNS absorption values lesser than one. Hence, the above compounds will not pass through BBB. The compounds which are having % plasma protein bounding (PPB) of less than 90% are weakly bounded, and % PPB greater than 90% indicates a strongly bounded compound. All the reported compounds have weak plasma protein binding. Caco-2 cell permeability of more than 70 indicates high permeability, and less than 4 indicates low permeability. A range between 4–70 indicates the intermediate permeability. The compounds ZINC000216155214 and ZINC000001643171 showed low Caco2 cell permeability of 0.32260 and 0.38902, respectively. MDCK permeability can be utilized to screen rapid permeability. The ligands ZINC1576020, ZINC000618254662, ZINC000216155214, ZINC000017064359 showed low permeability, (MDCK < 25) and ZINC000001704450 showed highest MDCK permeability 249.543 nm/s.

Table S2 shows the results of the toxicological properties of mutagenicity (Ames Test) and carcinogenicity (Mouse and rat). The Ames test is a simple method to test the mutagenicity of a compound, suggested by Ames. All the compounds were predicted as positive, which means they are mutagen. Carcinogenicity is the ability that a substance has to induce alterations that lead to cancer. All the compounds presented were negative predictions for carcinogenicity in mice, which means that there is evidence of carcinogenic activities. In the prediction of carcinogenicity in rats, all the compounds showed evidence of carcinogenic activities except ZINC000216155214, which showed positive, which means that show no carcinogenic activity.

### 3.6. PAINS Prediction

Many of the frequent hitters (show higher hit rates than expected or appear) compounds are PAINS, aggregators, or reactive compounds; however, notably, a substantial part of frequent hitters are actual promiscuous compounds. The ability to bind to multiple binding sites was related to “privileged scaffolds” or “master key compounds”, termed true promiscuity.

Hit Dexter web server predicted the six compounds as non-promiscuous by PSA (primary screening assays) classifier and CDRA (confirmatory dose-response assays) classifiers. Therefore, it demonstrates these compounds would be specific compounds rather than promiscuous ones (Table S3).

According to Swiss ADME online tool results, all compounds showed 0 alerts for PAINS, except ligand ZINC000618254662, which showed PAINS 1 alert.

## 4. Discussion

Type 2 diabetes mellitus (T2DM) is a common defect affecting millions of humans [31]. T2DM is characterized by insulin resistance in the hepatic and peripheral organs. The glucose transporter 4 (GLUT4) is important in the pathogenesis of T2DM. In T2DM patients, its faulty expression or translocation to the peripheral cell plasma membrane impedes glucose entry into the cell for energy production. When controlling the glucose metabolism of T2DM patients, in addition to appropriate medicines, an adequate diet and/or exercise can be used to target the increase in GLUT4 expression, GLUT4 concentrations, and GLUT4 translocation to the cell surface. Furthermore, as potential targets or molecules, some potentially good synthetic and natural drugs that can activate the insulin-independent GLUT4 signaling pathways for the efficient control of T2DM are emphasized [32]. Researchers are striving mutually for a better drug development technique and cure against T2DM [33,34]. Computational approaches were employed to analyze the effect of chemical compounds through virtual screening, molecular docking, and ADMET studies [35,36]. Recently, molecular docking analyses along with virtual screening were performed against the drug candidates in clinical trials and approved drugs [37,38].

Protein 3D structure prediction of GLLUT4 is performed comparatively using multiple approaches for homology modeling (Modeler) [39], for threading (Quark), and for Ab-initio (ITASSER) [40] was used. The best model is selected on the basis of its high-quality factor by analyzing the Ramachandran plot [41].

In this study, a virtual screening analysis has been carried out to identify potential drug candidates from an active phytochemical library comprising 5000 compounds. Multiple criteria were followed to screen 5000 compounds. First of all, compounds are screened on the basis of their respective binding poses and binding energies, i.e., compounds having the least binding energies are known to be more perfectly bound as compared to compounds having high binding energies [42]. In our study, the ligands for each complex were ranked using PyRx based on a precise prediction of position in order to separate those ligands that did not bind in a ranked list. The hits obtained from the virtual screening were subjected to molecular docking studies; the top two molecules with the highest docking scores were selected for the study of binding modes. have obtained excellent docking scores, which indicates that they have good interaction with the target and can inhibit the GLUT4 protein.

The second criterion which is followed was its RMSD value, as RMSD refers to the comparative distance between bounded atoms from ligand and receptor. RMSD has often been used to measure the quality of reproduction of a known (i.e., crystallographic) binding pose by a computational method, such as docking. RMSD for the best docking complex should be in the range of 1 to 3 Angstrom. Our studies show that ZINC000001576020 has the lowest RMSD 1.1 Å and ZINC000216155214 has the highest RMSD 20.0 Å. Both RMSDs are in the criteria range which shows the accuracy of our results.

Based on the docking score, ligand ZINC000216155214 docked with GLUT4 resulted in the highest docking score, which suggests the excellent interaction between ligand-target complex. The following are the amino acids responsible for their binding affinity [43].

In humans, the absorption, distribution, metabolism, excretion, and toxicity (ADMET) properties of a molecule can be affected by the arrangement in the system, which is connected at the molecular level by various units (transporters, channels, receptors, and enzymes). The earlier understanding of the binding interactions and predictions of toxicity can reduce the chances of drug failure in the final stage of drug development [42]. ADMET studies can also examine if the molecule binds to receptors that affect the regulation of other proteins and if it interferes with endogenous metabolic, regulatory proteins, and transport [44,45]. Therefore, ADMET properties were also calculated to check the drug ability and drug-likeness of selected compounds [46]. All compounds are non-toxic, evaluated by AdmetSAR, and have zero Lipinski’s rule of five violations [47].

HIA is an important parameter for the selection, optimization, and development of candidates for oral medication. The recommended range for the HIA is 70–100% well absorbed, 20–70% moderately absorbed, and 0–20% poorly absorbed. According to the results, six active compounds had good to moderate HIA values [48], which suggests that they possess good oral bioavailability. BBB penetration is the essential parameter in the drug discovery stages, as compounds that act on the central nervous system (CNS) must pass through it and have BBB predicted value greater than one, whereas inactive compounds will be less than one, which indicates that they do not pass through BBB. According to this, the six ligands were found to be inactive in the CNS [49]. The efficacy or biological activity of the drug depends on the degree of plasma protein bounding, which influences the half-life of the drug. The bounded portion of the drug is responsible for the biological action, whereas the unbounded form is metabolized and excreted from the body. Based on the PPB results, the compounds are found to bind weakly with plasma protein [50]. For the prediction of oral drug absorption, Madin–Darby canine kidney (MDCK) cells [51] and Caco-2 cell models [52] were considered. Caco-2 cells are a well-differentiated intestinal cell line derived from human colorectal carcinoma that display similar morphological and functional properties of the in-vivo intestinal epithelial cell barrier. The advantage of MDCK cells is having a shorter growth period compared to Caco-2 cells. All the ligands showed lower to moderate Caco-2 and MDCK permeability. ZINC000001643171 and ZINC000216155214 have low Caco-2 cell permeation. Thus, they have poor oral drug absorption, whereas ZINC1570006020 has good oral drug absorption.

Lipinski’s “Rule of 5” states that most “drug-like” molecules have log P < or =5, number of hydrogen bond acceptors < or =10, number of hydrogen bond donors < or =5, and molecular weight < or =500 g/mol. Molecules violating more than one of these rules may have problems with bioavailability [53]. In this study, all six ligands were found to have a molecular weight below 300, log P below 5, H-bond acceptors (ranged from 5 to 8) and donors (ranged from 1 to 4) were found to be within the permissible limit. Thus, they found no violations of Lipinski’s “Rule of 5”. This indicates that these compounds will have good oral bioavailability.

One of the important reasons for the discovery of new drugs is the evaluation of the toxicity of drug candidates. This means that the conception of drugs with consideration of their toxicity is very important, as well as predicting the mutagenicity and carcinogenicity of new compounds that may be toxic. These results suggest that selected compounds are more suitable to be used as drug candidates against T2DM as these compounds are highly targetable, non-toxic, and have very good binding affinities.

## 5. Conclusions

The aim of this work was to identify the effective chemical compounds against T2DM candidate protein glucose transporter 4. The predicted six chemical compounds that were screened leading to the molecular docking analyses against GLUT4 protein, and interactional analyses of the selected docked complexes were analyzed. In addition, their physicochemical and ADMET properties were predicted, which indicates that they are druggable with no Lipinski’s R05 violations and non-toxic in nature. Furthermore, they are predicted as non-promiscuous compounds. In conclusion, two active compounds (ZINC000216155214 and ZINC000001576020), were predicted as potential targets as inhibitors against T2DM.

## Figures and Tables

**Table 1:** List of the ligands with SMILES.

Ligand	Canonical_SMILES
ZINC000001576020	C[C@@H](NC(=O)[C@H]1C[C@H](O)CN1C(=O)CN)C(=O)O
ZINC000001643171	CN(/N=C/C=N/N(C)C1=NCCN1)C1=NCCN1
ZINC000001704450	CC(C)C[C@H](N)C(=O)O[C@H](N)C(=O)NCCN(C)C
ZINC000017064359	Cn1c(=O)c2[nH]c(-[n+]3cccc(CO)c3)nc2n(C)c1=O
ZINC000216155214	CN1CCN(C2=C3C(=O)NON3[C@@H]([N+](=O)[O-])C=C2)CC1
ZINC000618254662	O=C(O)CNC(=O)CN/C=C1\SC(=S)NC1=O

**Table 2:** Showing physicochemical properties and toxicity of all selected active chemical compounds by admetSAR.

Ligand	Molecular Weight (g/Mol)	Octanol−Water Partition Coefficient (LogP)	H-Donor	H-Acceptor	Lipinski Violation	Toxicity
ZINC000001576020	259.262	−2.5038	4	5	0	Non-toxic
ZINC000001643171	250.31	−1.26	2	8	0	Non-toxic
ZINC000001704450	274.365	−1.1344	3	6	0	Non-toxic
ZINC000017064359	288.287	−1.2707	2	6	0	Non-toxic
ZINC000216155214	281.272	−1.1036	1	7	0	Non-toxic
ZINC000618254662	275.311	−1.2339	4	6	0	Non-toxic

**Table 3:** Showing binding affinity, RMSD, and interacting residues of Top selected active chemical compounds.

Ligand	S-Score (Kcal/Mol)	RMSD (Å)	Interacting Residues
ZINC000216155214	−6.5	0.0	ASN304, GLU396, ASN431
ZINC000001576020	−5.9	1.1	ASN333, GLU396, ASN431

## Data Availability

Nucleotide sequence for this gene study is retrieved from the GenBank NCBI Database (https://www.ncbi.nlm.nih.gov/genbank/ accessed on 5 February 2022), and the protein sequence for this protein was retrieved from the UniProt knowledge database (https://www.uniprot.org accessed on 5 February 2022). A library of 5003 natural phytochemicals was created by obtaining compounds from different open-source databases such as ZINC, ChEMBL, and PubChem.

## Supplementary Materials

The following supporting information can be downloaded at: https://www.mdpi.com/article/10.3390/jpm13040660/s1, Table S1. ADME Properties by PreADMET tool; Table S2. Toxicity properties by PreADMET tool; Table S3. Prediction of PAINS by promiscuity assessments.
