# Supplementary material for: Elucidating the Potential Inhibitor against Type 2 Diabetes Mellitus Associated Gene of GLUT4
Source: J Pers Med. 2023 Apr 12;13(4):660. doi: 10.3390/jpm13040660 (PMC10146764; doi:10.3390/jpm13040660)
Supplement: Supplementary file 1 [file jpm-13-00660-s001.zip › jpm-2225864-supplementary.pdf]

Supplementary Tables

Table S1. ADME Properties by PreADMET tool

Ligand	%HIA	BBB	PPB%	Caco-2 (nm/s))	MDCK (nm/s))
ZINC1576020	24.605537	0.0563796	1.83723	21.0234	1.01374
ZINC000001643171	86.250532	0.0376253	0.000000	0.38902	4.29762
ZINC000001704450	56.751972	0.0333579	11.546380	15.745	249.543
ZINC000017064359	83.479537	0.0391296	11.217050	19.0693	0.621723
ZINC000216155214	79.395248	0.0161232	23.305426	0.3226	109.014
ZINC000618254662	62.614416	0.0627102	11.831642	15.7208	0.465821

Table S2. Toxicity properties by PreADMET tool

Ligand	AMES TEST	Carcinogenicity	
		Mouse	Rat
ZINC1576020	mutagen	negative	negative
ZINC000001643171	mutagen	negative	negative
ZINC000001704450	mutagen	negative	negative
ZINC000017064359	mutagen	negative	negative
ZINC000216155214	mutagen	negative	positive
ZINC000618254662	mutagen	negative	negative

Table S3. Prediction of PAINS by promiscuity assessments

Ligand	Probability of a compound being promiscuous PSA/ CDRA		Promiscuity Assessment		PAINS
	PSA	CDRA	PSA classifier	CDRA classifier	
ZINC000001576020	0.06	0.06	non-promiscuous	non-promiscuous	**0 alert**
ZINC000001643171	0.29	0.18	non-promiscuous	non-promiscuous	**0 alert**
ZINC000001704450	0.05	0.08	non-promiscuous	non-promiscuous	0
ZINC000017064359	0.12	0.25	non-promiscuous	non-promiscuous	0
ZINC000216155214	0.43	0.36	non-promiscuous	non-promiscuous	0
ZINC000618254662	0.18	0.43	non-promiscuous	non-promiscuous	1 alert
